# Non-coding RNAs regulating endothelial progenitor cells for venous thrombosis: promising therapy and innovation

**DOI:** 10.1186/s13287-023-03621-z

**Published:** 2024-01-02

**Authors:** Li-Li Sun, Zhao Liu, Feng Ran, Dian Huang, Ming Zhang, Xiao-Qiang Li, Wen-Dong Li

**Affiliations:** Department of Vascular Surgery, Nanjing Drum Tower Hospital, The Affiliate Hospital of Nanjing University Medical School, #321 Zhongshan Road, Nanjing, 210008 Jiangsu China

**Keywords:** Non-coding RNAs, Endothelial progenitor cells, Deep venous thrombosis, Regenerative medicine

## Abstract

Venous thromboembolism, which includes deep venous thrombosis (DVT) and pulmonary embolism, is the third most common vascular disease in the world and seriously threatens the lives of patients. Currently, the effect of conventional treatments on DVT is limited. Endothelial progenitor cells (EPCs) play an important role in the resolution and recanalization of DVT, but an unfavorable microenvironment reduces EPC function. Non-coding RNAs, especially long non-coding RNAs and microRNAs, play a crucial role in improving the biological function of EPCs. Non-coding RNAs have become clinical biomarkers of diseases and are expected to serve as new targets for disease intervention. A theoretical and experimental basis for the development of new methods for preventing and treating DVT in the clinic will be provided by studies on the role and molecular mechanism of non-coding RNAs regulating EPC function in the occurrence and development of DVT. To summarize, the characteristics of venous thrombosis, the regulatory role of EPCs in venous thrombosis, and the effect of non-coding RNAs regulating EPCs on venous thrombosis are reviewed. This summary serves as a useful reference and theoretical basis for research into the diagnosis, prevention, treatment, and prognosis of venous thrombosis.

## Introduction

Venous thromboembolism (VTE) includes deep venous thrombosis (DVT) and pulmonary embolism (PE), which is the complete or incomplete occlusion of blood vessels due to abnormal coagulation of blood in the veins [1]. The incidence of VTE is second only to acute coronary syndrome and stroke, and its prevalence significantly increases with age [2–5]. The estimated average annual incidence of overall VTE among people of European ancestry is 104–183 cases per 100,000 inhabitants [6], and the rate of recurrent VTE within 10 years after the initial event ranges from 20 to 36% [7]. Therefore, VTE is a common disorder that recurs frequently and is associated with significant morbidity and mortality.

The standard treatment for DVT is based on anticoagulation, which slows the further increase in venous thrombosis but does not reduce the incidence of post-thrombotic syndrome (PTS) and PE [8–10]. Additionally, anticoagulation treatment cannot accelerate the resolution and removal of formed venous thrombosis and repair venous valves, and it increases the risk of bleeding in patients [10–12]. Currently, the conventional treatments for subacute and chronic DVT and PTS are limited, and the treatment effect for some patients is not satisfactory [13–15]. Therefore, it is necessary to explore the new ideas and methods for prevention and treatment of DVT.

Asahara et al. [16] first successfully isolated endothelial progenitor cells (EPCs), as precursors of endothelial cells, from peripheral blood, and EPCs were confirmed to be undifferentiated cells involved in angiogenesis. Currently, express markers on the surface of EPCs are still controversial; however, the current international consensus is that EPCs exhibit a phenotype of CD45^−^CD34^+^VEGFR2^+^ or CD45^−^CD34^+^CD133^+^VEGFR2^+^ [17]. In recent years, studies have shown that EPCs participate in neovascularization and endothelial repair, and EPCs have emerged as a promising treatment for DVT-related diseases in patients with poor therapeutic effects in current treatment strategies [18, 19]. However, adverse conditions of the microenvironment, such as smoking, advanced age, diabetes, cardiovascular risk factors, ischemic diseases, and transplanted vascular diseases, affect the number and function of EPCs and reduce the effectiveness of EPCs in treating DVT [20–23]. Therefore, the development of methods to improve the recruitment of EPCs to the thrombus site and enhance the angiogenesis of EPCs is particularly critical for treating DVT.

Numerous studies have shown that non-coding RNAs play a major role in the biological function of EPCs and participate in regulating the occurrence and development of VTE [24, 25]. Additionally, non-coding RNAs have shown promising results in research on the treatment of VTE with EPCs [11, 26–28]. This review analyzes the characteristics of venous thrombosis, the effect of EPCs on venous thrombosis, and the regulatory role of non-coding RNAs regulating EPCs in venous thrombosis, providing an overview of the potential value of EPCs for the diagnosis and treatment of VTE.

## Characteristics of venous thrombosis

DVT is mainly due to venous endothelial damage, slow blood flow, and the hypercoagulable state of blood, which causes abnormal blood coagulation in deep veins and venous reflux disorders [29]. DVT mostly occurs in deep veins of the lower extremities [18]. There are approximately 900,000 new cases of DVT each year in the USA, with nearly 300,000 DVT-related deaths annually [30]. DVT can cause acute PE, which threatens the life of patients, and chronic thromboembolic pulmonary hypertension [31–33]. Approximately 3% of patients with symptomatic PE develop chronic thromboembolic pulmonary hypertension within 2 years, which can be fatal [34–36]. PTS is often caused by chronic venous obstruction and reflux in the stage of chronic DVT. Approximately 20–50% of patients with DVT develop PTS after anticoagulation therapy, of which 5–10% are severe, leading to edema, pain, heaviness, pigmentation, lipid sclerosis, and even venous ulcers in the affected extremity [1, 37]. The patient’s quality of life and survival are seriously affected by these symptoms [38, 39].

Non-surgical treatment based on drug therapy cannot effectively improve the patency rate of the venous lumen and cannot reduce the recurrence rate. Furthermore, this treatment is time-consuming and labor-intensive, and the effectiveness of medication is often unsatisfactory. Although surgical treatment has a short treatment period, it is expensive, and there is a risk of bleeding, infection, and recurrence, which increase the physical and economic burden of patients. Additionally, some older or critical patients are not suitable for surgery because of their physical condition. Currently, conventional treatment methods have disadvantages, such as an easy relapse, long treatment cycle, high cost, and unsatisfactory curative effect, which seriously threaten the economic status and mental health of patients [40]. Studies have shown that recurrence, PTS, hemorrhage caused by anticoagulation, and death are the main adverse consequences of DVT [32, 41]. In patients with DVT, especially those with PTS, their quality of life is severely affected [32, 42]. In summary, these are difficult problems in the treatment of DVT in clinical work. Therefore, the new prevention and treatment approach for DVT needs to be investigated. Patients with DVT must have an early diagnosis, risk assessment, early treatment, and prognostic evaluation without damaging the physiological hemostatic system to avoid the serious or even fatal consequences of thromboembolism, such as acute PE. The discovery of accurate biomarkers is difficult because of the lack of specificity in the clinical manifestations of VTE. Therefore, further study on the molecular mechanisms underlying the occurrence and development of DVT, exploring new biological treatment methods, and identifying accurate biomarkers for early diagnosis and safe, efficient, and precise targets for prognostic evaluation have important clinical value. This understanding will lead to new prospects for the advancement of diagnosis of clinical disease and treatment technique.

## Role of EPCs in venous thrombosis

**Fig. 1:**
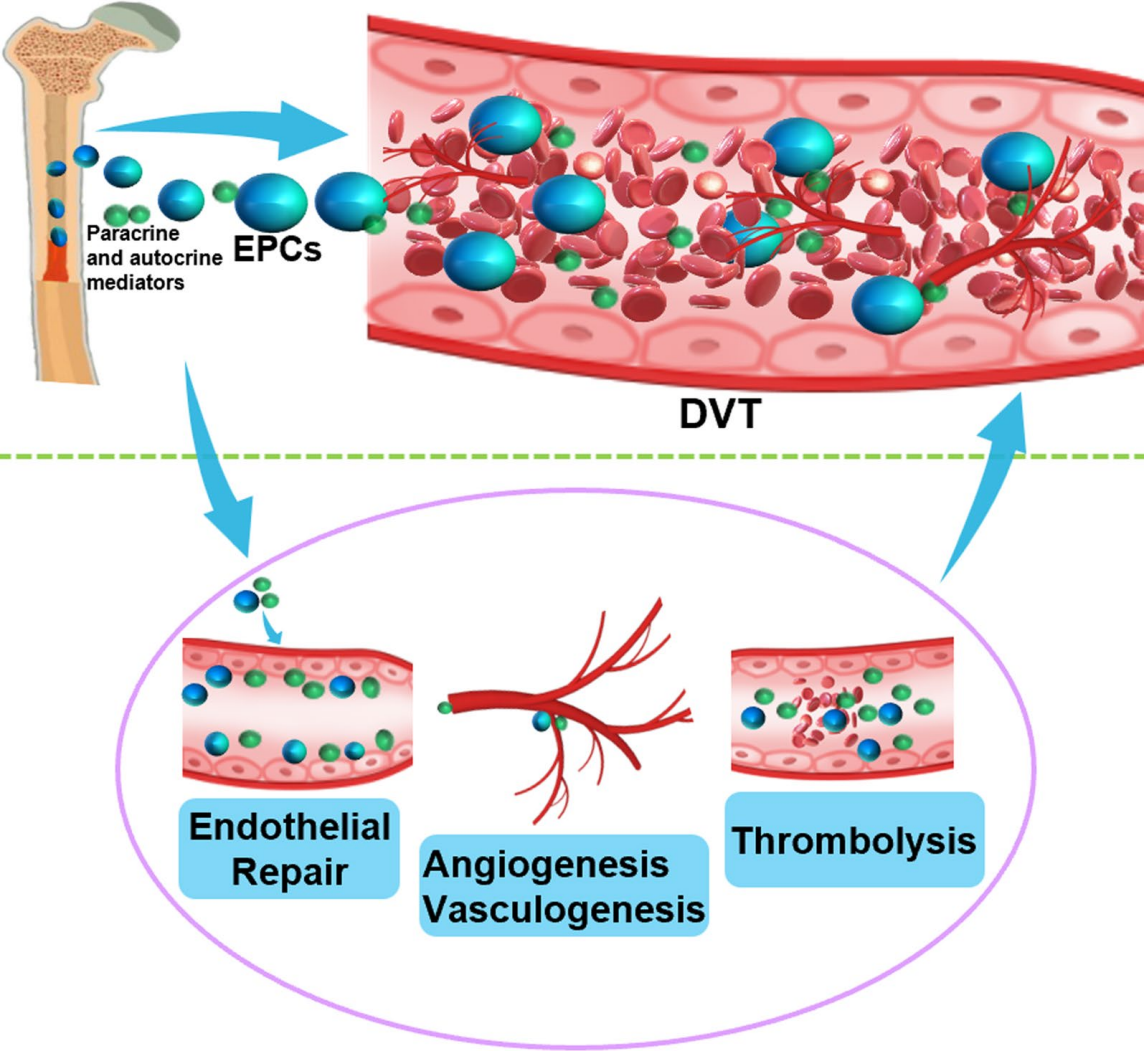
The role of endothelial progenitor cells in deep vein thrombosis. Endothelial progenitor cells migrate and recruit to the site of venous thrombosis to accelerate the resolution, organization, and recanalization of DVT through endothelial repair, angiogenesis, and thrombolytic factors by paracrine and autocrine pathways

EPCs not only can proliferate, migrate, and form new blood vessels through differentiation into endothelial cells but also secrete cytokines and vascular growth factors, which play an important role in the process of endothelial repair and angiogenesis [16, 18]. Accordingly, they have promising potential in the biological treatment of DVT. Additionally, EPCs are recruited to the thrombus site through the secretion of vascular growth factors, cytokines, and other factors to accelerate the resolution and recanalization of the thrombus [18]. By using a murine DVT model, a previous study has demonstrated that the number of intrathrombotic EPCs changes dynamically with the advance of thrombus age [43]. In detail, EPCs were initially observed at the thrombus age of 5 days, and the number of EPCs was the largest in the 10-day thrombus [43]. The results suggested the practicality of EPCs as a marker for thrombus age determination. Figure 1 illustrates their detailed characteristics in DVT, that is, EPCs migrate and recruit to the site of venous thrombosis to accelerate the resolution, organization, and recanalization of DVT through endothelial repair, angiogenesis, and thrombolytic factors.

Moreover, EPCs also play an important role in physiology and pathological angiogenesis in adults [44–47], and they have become a promising treatment method for DVT-related diseases in patients in whom current conventional strategies are not effective [46–48]. Currently, EPCs have become an active topic, and their position in regenerative medicine has become increasingly prominent. In vascular tissue engineering and cell therapy, especially in the treatment of vascular diseases, they have shown great potential clinical application value. However, although EPCs have shown their potential therapeutic value, their clinical application still faces many challenges. A variety of adverse conditions in the microenvironment, including smoking, advanced age, diabetes, cardiovascular risk factors, ischemic diseases, and graft vascular disease, affect the number and function of EPCs [27, 49–51]. Therefore, the development of ideas and methods to improve the recruitment of EPCs to the thrombus site and enhance angiogenesis is of great significance for the treatment of DVT using EPCs.

## Regulatory effects of non-coding RNAs of EPCs involved in venous thrombosis

High-throughput sequencing technology, epigenomics, gene prediction technology, and bioinformatics have developed rapidly in recent years. The total amount of newly discovered functional non-coding RNAs has also been rising [52]. In particular, studies on long non-coding RNAs (lncRNAs), microRNAs (miRNAs), and circular RNAs (circRNAs) and their functions and mechanisms have become comprehensive, in-depth, and detailed.

### Biology of non-coding RNAs

#### Biology of lncRNAs

LncRNAs belong to a class of non-coding RNAs with more than 200 nucleotides, and they function mainly through epigenetic modification and regulation of transcription and translation [53]. Studies have shown that lncRNAs can act as miRNA competitors or sponge molecules to indirectly regulate gene expression [54, 55]. Furthermore, lncRNAs are important regulators in angiogenesis, development, differentiation, metabolism, and autophagy [27, 56]. Many lncRNAs are dysregulated in a variety of diseases, including vascular diseases, and they participate in the regulation of disease progression [26, 57, 58]. LncRNAs can be used as potential targets and biological markers for the diagnosis and prognosis of disease and serve as new targets for the treatment of vascular diseases. Recent studies have shown that lncRNA expression is unbalanced in EPCs in patients with venous thrombosis [26, 27]. This in turn regulates the biological functions of EPCs that participate in the resolution and recanalization of VTE.

#### Biology of miRNAs

MiRNAs are conserved non-coding single-stranded molecules that are composed of approximately 22 nucleotides [59]. These small RNAs inhibit the translation of target mRNAs or regulate their degradation by targeting the 3' untranslated region [60–63]. In the process of angiogenesis, they play an irreplaceable role in regulating the proliferation, differentiation, apoptosis, migration, and angiogenic ability of angiogenesis-related cells. This activity plays an important role in a variety of physiological and pathological processes, especially in the occurrence and development of vascular diseases.

#### Biology of circRNAs

CircRNAs, a new type of non-coding single-stranded RNAs with a conserved circular structure, are indigestible by RNases due to the lack of the 5′ cap and 3′ poly(A) tail [64]. CircRNAs play a regulatory role through various mechanisms, including acting as miRNA sponges and regulating alternative splicing, parental gene expression, and protein translation, and have been found to play a significant role in vascular diseases in recent years [65–67].

To sum up, non-coding RNAs have important effects on vascular diseases. An increasing number of studies have shown that the expression of lncRNAs, miRNAs, and/or circRNAs is unbalanced in a variety of diseases, which may lead to abnormal angiogenesis, thereby regulating the occurrence and progression of disease [11, 24, 26, 58]. Therefore, non-coding RNAs can be used as potential biomarkers and intervention targets for the diagnosis, treatment, and prognosis of diseases [68–70].

### Role and mechanism of non-coding RNAs in regulating EPCs in venous thrombosis

#### Role and mechanism of lncRNAs in regulating EPCs in venous thrombosis

Compared with cancer and cardiovascular diseases, there are few studies on lncRNA in DVT. Fortunately, a previous study found that LINC00659 expression was significantly elevated in the peripheral blood of DVT patients at high altitudes compared with high-altitude controls by using RNA sequencing technology [71]. Mechanistically, LINC00659 and UXT-AS1 compete with miR-143 and miR-15 through their miRNA response elements, thereby regulating the expression of *HIF1A*, *NRG1*, *FLT1*, *SERPINE1*, and *FGF1* [71]. Recently, LINC00659 expression has also been found to be increased in inferior vena cava tissues and peripheral blood of lower extremity DVT, and overexpression of LINC00659 inhibits EPC migration, proliferation, and angiogenesis by activating DNMT3A-mediated *FGF1* promoter methylation [72]. And inhibition of LINC00659 prevents lower limb DVT progression, as shown using a DVT mouse model [72]. These studies suggest that LINC00659 is a potential biomarker and intervention target of DVT. Additionally, a study has shown that the lncRNA metastasis-associated lung adenocarcinoma transcript 1 (MALAT1) is upregulated in DVT samples compared with healthy controls, and it inhibits the proliferation and migration of EPCs in DVT by regulating the Wnt/β-catenin pathway [73]. This finding suggests that the MALAT1/Wnt/β-catenin pathway is a new target for DVT treatment. Moreover, a study showed that the expression levels of lncRNA sirtuin 1 (Sirt1)-antisense (AS) and *Sirt1* mRNA in the blood of patients with DVT were significantly decreased [74]. And lncRNA Sirt1-AS upregulates Sirt1 in human vascular endothelial cells and reduces the expression of biomarkers related to senescence and DVT through FOXO3a ubiquitination and degradation [74]. This study suggested that lncRNA Sirt1-AS may be a potential new biomarker of DVT.

The interaction of non-coding RNAs, especially lncRNAs and miRNAs, forms a complex regulatory network and participates in the occurrence and development of many diseases [75]. Studies have reported that the lncRNA–miRNA–mRNA axis plays an important role in vascular diseases [76, 77]. Animal experiments have shown that knockout of lncRNA ANRIL reduces thrombosis in rats [78]. Mechanistically, ANRIL upregulates autophagy to promote angiogenesis and thrombosis by targeting miR-99a and miR-449a expression. Our team showed that the lncRNA Wilms tumor 1-associated protein pseudogene 1 (WTAPP1) was significantly downregulated in EPCs in patients with DVT [79], and we further found that WTAPP1 overexpression regulates matrix metalloproteinase-1 through miR-3120 and the Akt/PI3K/autophagy pathway to promote EPC migration and angiogenesis [27].

In addition, a previous study showed that the expression levels of the lncRNAs NR_036693, NR_027783, NR_033766, and NR_001284 in arterial endothelial tissue in patients with chronic thromboembolic pulmonary hypertension were significantly changed [80]. These lncRNAs are mainly involved in inflammation, endogenous stimulus, and antigen presentation. This finding provides experimental evidence for the diagnosis and treatment of chronic thromboembolic pulmonary hypertension. Additionally, studies have shown that some drugs participate in the regulation of disease progression by regulating non-coding RNA [81]. Recent studies have shown that by regulating the MALAT1/miR-22-3p/NLRP3 signaling pathway, resveratrol inhibits the activation of inflammasomes, thereby reducing heart injury related to PE [81].

The number of functional lncRNAs discovered is continuously increasing. The functions and mechanisms of lncRNAs have become more comprehensive, detailed, and in-depth with this increase in knowledge. This knowledge will help to fully reveal the details of gene expression regulation and help provide a deeper understanding of the occurrence and development of VTE. New opportunities and ideas for the prevention, early diagnosis, treatment, and prognostic evaluation of VTE are provided by research into its mechanism.

#### Role and mechanism of miRNAs in regulating EPCs in venous thrombosis

As the precursor cells of endothelial cells, EPCs can migrate to peripheral blood and differentiate into mature endothelial cells, and many miRNAs regulate this process. Dysregulation of these miRNAs leads to functional disorders of EPCs, thereby regulating the occurrence and development of DVT [18].

A study showed that miR-483-3p, which is upregulated in patients with DVT, downregulated serum response factor, thereby reducing EPC migration and angiogenesis and promoting apoptosis [82]. Our previous study showed that miR-205 overexpression in EPCs targeted PTEN, downregulated PTEN mRNA and protein expression levels, and regulated the expression of matrix metalloproteinase-2 through the AKT/autophagy pathway, thus enhancing the migration, invasion, proliferation, and angiogenesis abilities of EPCs and further accelerating the resolution and recanalization of DVT [11]. These findings provide a new direction and experimental basis for the development of new treatment methods for DVT. Similarly, miR-9 in EPCs targets TRPM7 and plays a major regulatory role in the migration, invasion, proliferation, and angiogenesis of EPCs through the PI3K/AKT/autophagy pathway [28]. The resolution and recanalization of DVT are accelerated by EPCs overexpressing miR-9 [28]. Recent studies have shown that miR-21 expression levels are reduced and that the resolution and recanalization of venous thrombosis in animal models of DVT are promoted through the injection of EPCs overexpressing miR-21 [83]. Mechanically, miR-21 overexpression significantly promotes the proliferation, migration, and angiogenic abilities of EPCs through targeting FASLG [83]. FAS combines with its ligand FASLG to transmit apoptotic signals, thereby regulating apoptosis [84]. Importantly, studies have shown that serum miR-21 levels in patients with DVT are reduced, which is closely related to an increase in recurrent DVT and PTS [83]. Therefore, miR-21 may be an independent risk factor for predicting the recurrence of DVT. Additionally, the proliferation of EPCs is closely related to miR-150, which controls the expression of SRC kinase signaling inhibitor 1 [85]. The recanalization of venous thrombosis in animal models of DVT is promoted by overexpression of miR-150 and the accompanying decrease in SRC kinase signaling inhibitor 1 expression levels [85]. In vitro studies have shown that angiogenesis and proliferation of EPCs are promoted through miR-150 overexpression [85]. Additionally, a previous study has shown that the function of EPCs can be regulated by miR-126 directly targeting the *PIK3R2* gene by PI3K/Akt pathway [86]. Overexpression of miR-126 enhances the migration and tube formation ability of EPCs in vitro and promotes homing and venous thrombolysis of EPCs in vivo [86]. Furthermore, upregulation of miR-204-5p promotes the thrombolysis of DVT by accelerating EPC migration, invasion and angiogenesis through targeting *SPRED1* in rats [87]. In addition, miR-206 knockdown increases GJA1 expression to suppress autophagy in EPCs and enhance EPC proliferation, migration, and angiogenesis, thereby facilitating the homing of EPC to the thrombus site and enhancing thrombus resolution in DVT mouse models [88]. A recent study found that inhibition of miR-125a-5p enhances EPC migration and angiogenesis through upregulation of MCL-1, thereby accelerating EPC homing to thrombi and promoting thrombus resolution in mice [89]. Moreover, inhibition of miR-195 in EPCs regulates GABA_A_ receptor-related protein 1 and further promotes the proliferation and angiogenesis of EPCs [47]. A study showed that high miR-195-5p levels in the blood of patients with DVT are directly related to low B-cell lymphoma 2 expression [90]. Therefore, miR-195-5p may participate in the occurrence and development of DVT by regulating the apoptosis of endothelial cells [91]. Taken together, these studies suggest that miRNAs play a crucial role in modulating EPC function and thereby regulating the occurrence and progression of DVT.

Studies have shown that some drugs and/or exosomes regulate the progression of DVT by regulating miRNAs. Examples of this regulation are as follows. By regulating miR-542-3p to target angiopoietin-2, resveratrol enhances the angiogenesis of EPCs [92]. Metformin inhibits the angiogenesis of EPCs by reducing the expression of p27 and autophagy by reducing miR-221 expression [93]. Additionally, the angiogenesis and migration of EPCs are enhanced by miR-126 exosomes [94], thereby regulating the recanalization of DVT. A recent study has shown that miR-136-5p from EPC-released extracellular vesicles facilitates the dissolution of DVT by inhibiting *TXNIP* expression, providing a promising treatment target for DVT [95].

#### Role and mechanism of circRNAs in regulating EPCs in venous thrombosis

CircRNAs are a class of non-coding RNAs discovered in recent years. They play important roles in the diagnosis and treatment of cardiovascular diseases and tumors. However, there are few studies on the roles of circRNAs in DVT and the underlying mechanisms. A recent study found that hsa_circ_0001020 expression was upregulated in the peripheral blood of DVT patients, and it promoted EPC migration, invasion, and homing by targeting miR-29c-3p to increase the expression of *MDM2* [66]. Additionally, thrombus formation in vivo was reduced through hsa_circ_0001020 inhibition [66]. Therefore, hsa_circ_0001020 might be a potential diagnostic biomarker and therapeutic target for DVT.

## Conclusions

**Fig. 2:**
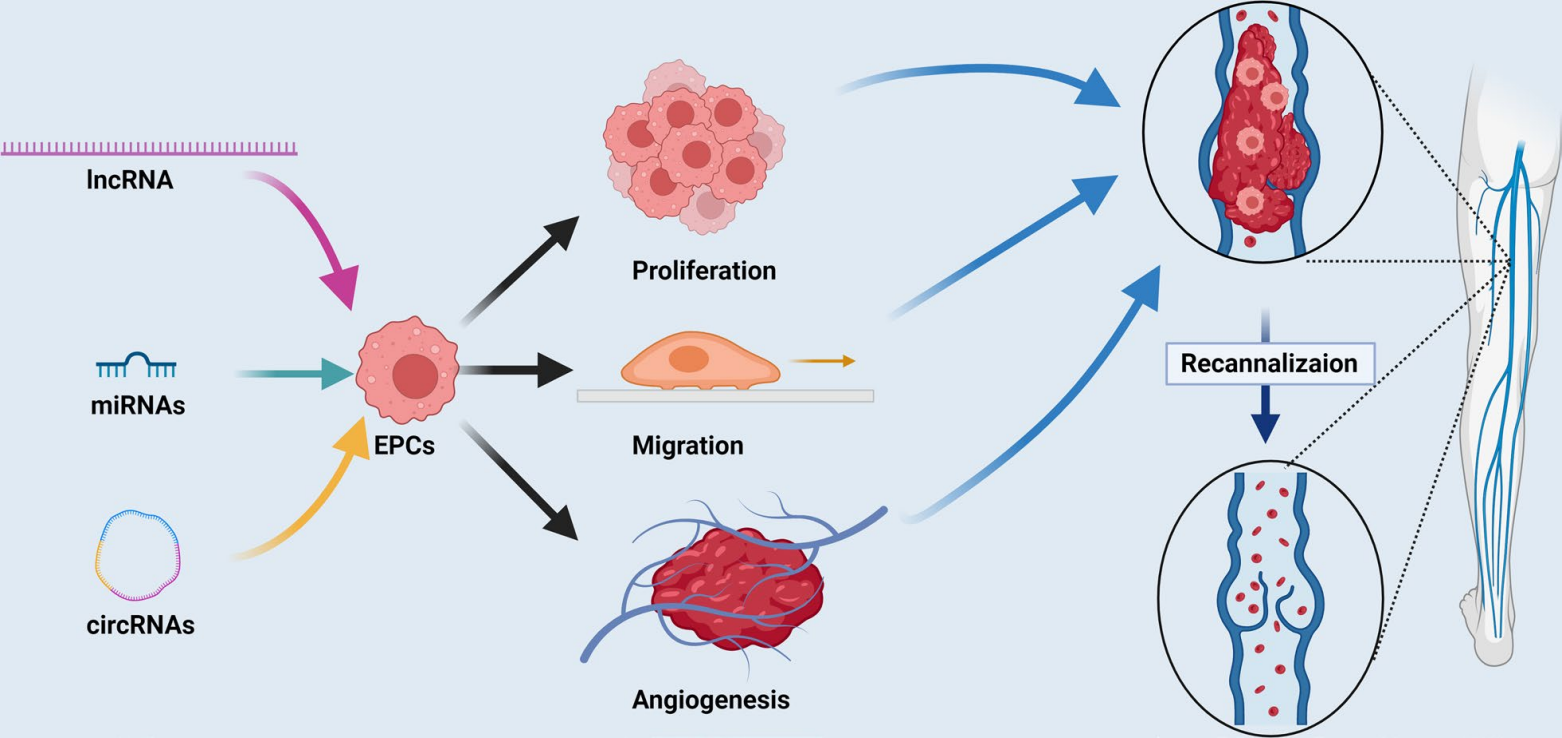
A schematic overview of non-coding RNA regulation of endothelial progenitor cells in the occurrence and development of deep vein thrombosis. LncRNAs, miRNAs and circRNAs participate in DVT resolution and revascularization by modulating EPC proliferation, angiogenesis, migration, and invasion, thus regulating the occurrence and progression of DVT, and non-coding RNA can be regarded as a potential marker and therapeutic target for DVT

Non-coding RNAs play an important regulatory role in EPCs in the occurrence and development of DVT. Generally, as shown in Fig. 2, lncRNAs, miRNAs, and circRNAs participate in DVT resolution and revascularization by modulating the proliferation, angiogenesis, migration, and invasion of EPCs, thus regulating the occurrence and progression of DVT, and non-coding RNA can be regarded as a potential marker and therapeutic target for DVT. These non-coding RNAs show great potential for clinical application regarding the diagnosis, treatment, prevention, and prognostic evaluation of DVT (Table 1). Their regulatory role in the outcome of disease is difficult to comprehensively study because of the complex regulatory network of the lncRNA–miRNA–mRNA axis. Therefore, future studies need to carefully and comprehensively verify non-coding RNAs related to disease pathology, but consistency between different patients should not be expected. Most researchers have focused on the functional role of a single RNA entity. However, studies have shown that there are complex interactions between different RNA molecules. To understand the interactions between different non-coding RNA species in mediating specific phenotypes, the use of network methods and advanced computational tools may be necessary. This could lead to understanding the function of non-coding RNAs in mediating disease phenotypes.

**Table 1:** Summary of non-coding RNAs regulation of endothelial progenitor cells involved in deep vein thrombosis

miRNA	Regulated target(s)/pathway	Function and main role in EPCs of DVT	References
LINC00659	miR-143, miR-15	Inhibition of EPC migration, proliferation, and angiogenesis	[71, 72]
MALAT1	Wnt/β-catenin pathway	Inhibition of EPC migration, proliferation	[73]
ANRIL	miR-99a,miR-449a	Promote EPC angiogenesis	[78]
WTAPP1	miR-3120	Promote EPC migration and angiogenesis	[27]
miR-483-3p	serum response factor	Inhibit EPC migration and angiogenesis and promote EPC apoptosis	[82]
miR-205	PTEN	Promote EPC migration, invasion, proliferation, and angiogenesis	[11]
miR-9	TRPM7	Promote EPC migration, invasion, proliferation, and angiogenesis	[28]
miR-21	FASLG	Promote EPC migration, proliferation, and angiogenesis	[83]
miR-150	SRC kinase signaling inhibitor 1	Promote EPC proliferation, and angiogenesis	[85]
miR-126	PIK3R2	Promote EPC migration, proliferation, and angiogenesis	[86]
miR-204-5p	SPRED1	Promote EPC migration, invasion, and angiogenesis	[87]
miR-206	GJA1	Inhibition of EPC proliferation, migration, and angiogenesis	[88]
circ_0001020	miR-29c-3p	Promote EPC migration, invasion, and homing	[66]

Future research directions should focus on the following: (1) how to accurately deliver active drugs to the site of venous thrombosis, improve the affinity of non-coding RNAs to target genes, and improve circulatory stability; (2) how to better evaluate the efficacy and response duration; (3) off-target effects; (4) the pharmacokinetic and pharmacodynamic properties of the molecules used; (5) the changes in the expression levels of various non-coding RNAs in EPCs during the occurrence and development of VTE, their in-depth regulatory effects, the underlying mechanisms, and their mutual regulation network; and (6) the role of non-coding RNAs in different cells in the VTE microenvironment and their interactions with inflammatory immune cells. Solving these important issues is important for the safe and effective clinical application of non-coding RNA-regulated EPCs. Additionally, understanding of these issues could lead to important changes in the diagnosis, treatment, and prognostic evaluation of VTE in the clinic. In summary, much research is required before non-coding RNA-regulated EPCs can be safely and effectively applied in the clinic. There needs to be further in-depth study of the molecular mechanism underlying the occurrence and development of VTE, as well as more clinical and basic research. New strategies using non-coding RNAs for the diagnosis and treatment of VTE expressed by stem cells have considerable clinical application value.

## Data Availability

All data are available on request.

## Abbreviations

DVT: Deep venous thrombosis
VTE: Venous thromboembolism
EPCs: Endothelial progenitor cells
PE: Pulmonary embolism
PTS: Post-thrombotic syndrome
LncRNAs: Long non-coding RNAs
MiRNAs: MicroRNAs
CircRNAs: Circular RNAs
WTAPP1: Wilms tumor 1-associated protein pseudogene 1
